# Scaling up Quality Improvement for Surgical Teams (QIST)—avoiding surgical site infection and anaemia at the time of surgery: a cluster randomised controlled trial of the effectiveness of quality improvement collaboratives to introduce change in the NHS

**DOI:** 10.1186/s13012-022-01193-9

**Published:** 2022-03-12

**Authors:** Ashley Brian Scrimshire, Alison Booth, Caroline Fairhurst, Elizabeth Coleman, Ajay Malviya, Alwyn Kotze, Chris Tiplady, David Tate, Annie Laverty, Gillian Davis, Win Tadd, Belen Corbacho, David J. Torgerson, Catriona McDaid, Mike Reed, the QIST collaborators, Mark Burgess, Gail Lowdon, Allan Marriott, Sue Cadwallader, Kathryn McLoughlin, Soha Sajid, Raza Hassan, Sharad Bhatnagar, Marshall Sangster, Hemant Sharma, Richard Holleyman, Rory Morrison

**Affiliations:** 1 Northumbria Healthcare NHS Foundation Trust, Woodhorn Lane, Ashington, NE63 9JJ UK; 2 York Trials Unit, Department of Health Sciences, University of York, York, YO10 5DD UK; 3 Leeds Teaching Hospitals, Leeds, UK; 4 Cardiff, UK

**Keywords:** Quality improvement collaborative, Cluster, Preoperative anaemia, MSSA, Decolonisation, Hip replacement, Knee replacement, *Staphylococcus aureus*

## Abstract

**Background:**

The aim of this trial was to assess the effectiveness of quality improvement collaboratives to implement large-scale change in the National Health Service (NHS) in the UK, specifically for improving outcomes in patients undergoing primary, elective total hip or knee replacement.

**Methods:**

We undertook a two-arm, cluster randomised controlled trial comparing the roll-out of two preoperative pathways: methicillin-sensitive *Staphylococcus aureus* (MSSA) decolonisation (infection arm) and anaemia screening and treatment (anaemia arm). NHS Trusts are public sector organisations that provide healthcare within a geographical area. NHS Trusts (*n* = 41) in England providing primary, elective total hip and knee replacements, but that did not have a preoperative anaemia screening or MSSA decolonisation pathway in place, were randomised to one of the two parallel collaboratives. Collaboratives took place from May 2018 to November 2019. Twenty-seven Trusts completed the trial (11 anaemia, 16 infection). Outcome data were collected for procedures performed between November 2018 and November 2019.

Co-primary outcomes were perioperative blood transfusion (within 7 days of surgery) and deep surgical site infection (SSI) caused by MSSA (within 90 days post-surgery) for the anaemia and infection trial arms, respectively. Secondary outcomes were deep and superficial SSIs (any organism), length of hospital stay, critical care admissions and unplanned readmissions. Process measures included the proportion of eligible patients receiving each preoperative initiative.

**Results:**

There were 19,254 procedures from 27 NHS Trusts included in the results (6324 from 11 Trusts in the anaemia arm, 12,930 from 16 Trusts in the infection arm). There were no improvements observed for blood transfusion (anaemia arm 183 (2.9%); infection arm 302 (2.3%) transfusions; adjusted odds ratio 1.20, 95% *CI* 0.52–2.75, *p* = 0.67) or MSSA deep SSI (anaemia arm 8 (0.13%); infection arm 18 (0.14%); adjusted odds ratio 1.01, 95% *CI* 0.42–2.46, *p* = 0.98). There were no significant improvements in any secondary outcome. This is despite process measures showing the preoperative pathways were implemented for 73.7% and 61.1% of eligible procedures in the infection and anaemia arms, respectively.

**Conclusions:**

Quality improvement collaboratives did not result in improved patient outcomes in this trial; however, there was some evidence they may support successful implementation of new preoperative pathways in the NHS.

**Trial registration:**

Prospectively registered on 15 February 2018, ISRCTN11085475

**Supplementary Information:**

The online version contains supplementary material available at 10.1186/s13012-022-01193-9.

Contributions to the literature

- Quality improvement collaboratives are increasingly used to implement change in healthcare; however, there are few randomised controlled trials on their effectiveness to improve patient- or provider-level outcomes.
- Collaboratives may be effective for implementing large-scale change in the NHS; however, changes in institutional behaviour may not lead to improvements in clinical outcomes.
- Future trials should expect a high early dropout rate and develop a recruitment strategy to mitigate against this. Staggered implementation of collaboratives so additional sites can be recruited later may help.
- Implementing new preoperative pathways using collaboratives in the NHS may take 9–10 months.

## Introduction

There are known gaps between what evidence shows to be best practice and the care that patients receive. The reasons for this are complex and multifactorial, and efforts to improve quality show mostly inconsistent and patchy results [1–3]. Such evidence-to-practice gaps exist in the fields of preoperative anaemia screening and optimisation, and preoperative methicillin-sensitive *Staphylococcus aureus* (MSSA) (a *S. aureus* bacterium) decolonisation, before major surgery. These practices have been recommended by multiple national and international guidelines and consensus statements since 2015/2016 and have been the focus of previous improvement programmes in the United Kingdom (UK) [4–11]. Yet despite this, the uptake of preoperative anaemia optimisation remains low, and considerable variability exists in *S. aureus* decolonisation practices in the UK [4, 6, 10, 12] and other European countries [13–16]. As such, an alternative approach to the implementation of these guidelines is warranted.

The development and implementation of clinical guidelines are intended to improve the quality, outcomes and cost-effectiveness of patient care [17]. However, as these two examples highlight, the implementation of clinical guidelines is challenging. The following barriers to guideline implementation have been identified in previous systematic reviews: guidelines that are complex to implement, healthcare professionals lack awareness or understanding, organisational constraints and a lack of resources or collaboration. However, implementation strategies that (i) are multifaceted and adequately resourced; (ii) incorporate guideline dissemination, education, audit and feedback; and (iii) actively engage clinicians and improve multi-professional collaboration are more likely to succeed [17–22].

Qualitative work specifically focussing on the barriers and facilitators to the implementation of preoperative anaemia optimisation pathways has reported similar findings [14, 23–26].

Quality improvement programmes provide a framework to bridge the evidence-to-practice gap. One increasingly popular technique is the quality improvement collaborative (QIC). The Institute for Healthcare Improvement developed the Breakthrough Series Collaborative methodology to design and deliver QICs [27, 28]. Whilst the clinical process, pathway or outcome being targeted and the setting in which they are implemented can vary, the overarching aim of QICs is to introduce change at scale by encouraging collaboration between teams from within and between healthcare organisations. The QIC methodology incorporates many of the factors identified as contributing to a successful implementation strategy. For example, a QIC involves a series of educational meetings (learning events) during which details of evidence-based changes and guidelines are disseminated and training and education are provided from a panel of expert faculty. These meetings actively engage clinician- and management-led multi-professional implementation teams from participating organisations, and networking and collaborative working is encouraged. In the time between these meetings (action periods), teams work on implementing changes in their local setting, taking into consideration the particular needs and local environment in which they operate. The use of initial small-scale, pilot changes is encouraged, and a programme of data collection (audit) and feedback should be embedded to monitor and evidence change [28].

A systematic review of 24 studies, including five randomised controlled trials (RCTs), found that QICs can be effective at implementing change in healthcare, particularly for improving process of care measures [29]. However, the review authors highlighted the need for more RCTs to assess the effects of QICs on provider- and patient-level outcomes. In particular, the review identified a lack of evidence on whether the procedural improvements associated with collaboratives translate into improved patient outcomes.

A more recent systematic review of 64 studies (10 RCTs, 24 before-after studies and 30 interrupted time series studies) on the effectiveness of QICs found that 83% of published studies reported a statistically significant improvement in at least one process or patient outcome measure, suggesting QICs may be an effective approach for introducing change in healthcare [30]. However, the authors highlighted an on-going need to address significant, persistent gaps in the reporting of QIC interventions and the quality of QIC trial design, noting a lack of high-quality RCTs. In addition, they suggested there is likely publication bias whereby QICs with negative findings are less likely to be published than those with positive findings.

The existing evidence demonstrates the feasibility and potential effectiveness of QICs but highlights the need for high-quality RCTs to assess their effects on processes of care, and provider- and patient-level outcomes [29].

This trial aims to assess the effectiveness of QICs to implement large-scale change in the UK National Health Service (NHS), specifically for improving outcomes in patients undergoing elective total hip replacement (THR) and total knee replacement (TKR). To achieve this, we compared the roll-out of two different preoperative initiatives to improve postoperative outcomes: anaemia screening and treatment (QIST: Anaemia) and MSSA nasal decolonisation (QIST: Infection). Both initiatives are associated with improved postoperative outcomes and are recommended by multiple national and international guidance, including those from the World Health Organisation (WHO) and the National Institute for Health and Care Excellence [6, 7, 31–37]. As the initiatives target healthcare teams, a cluster RCT was used.

## Methods

### Trial design, participants and outcomes

Methodology for the QIST cluster trial is detailed in the prospectively published protocol [38]. In summary, 41 volunteer NHS Trusts (public healthcare organisations serving a particular geographical area) that confirmed at the time of recruitment that they had neither a preoperative anaemia screening nor MSSA decolonisation pathway already in place for adult patients undergoing primary, elective THR or TKR were randomised (1:1) as clusters to join one of two parallel QICs. Such a design deals with the ‘Hawthorne effect’ by giving both groups an intervention which should abolish any effect due simply to greater interest in the hospital by external researchers. Group allocation, performed by the trial statisticians, was via minimisation with the number of THR and TKR procedures performed in 2016/2017 (≤660, >660) and the indicators in the Learning from Mistakes (LfM) league table (outstanding, good, significant concerns or poor) as minimisation factors. Trusts randomised to the QIST: Anaemia collaborative worked on implementing preoperative anaemia screening and optimisation pathways, expected to reduce perioperative blood transfusions within 7 days pre- or post-surgery (co-primary outcome). The QIST: Infection collaborative worked on implementing preoperative MSSA nasal decolonisation pathways, expected to reduce the incidence of postoperative deep surgical site infection (SSI) caused by MSSA within 90 days post-surgery (co-primary outcome), deep SSI caused by other organisms and superficial SSIs within 30 days of surgery (secondary outcomes). Critical care admissions, length of hospital stay and unplanned readmissions were secondary outcomes for both arms.

Implementation of the preoperative pathways at each site was measured by considering the proportion of the total number of THR or TKR procedures performed each month that received preoperative anaemia screening or MSSA screening and/or decolonisation as appropriate.

The two collaborative groups were kept separate with all QIC activities being held on different dates. As the two groups were focusing on different pathways, expected to improve different postoperative outcomes, the two arms of the trial acted as each other’s control. Due to the nature of the intervention, it was not possible to blind Trusts or treating clinicians to the trial arm they had been randomised to.

It was estimated that 30,000 procedures from 40 participating Trusts would provide 80% power to detect a difference from 0.75 to 0.25% in MSSA infection rate in favour of the infection arm and over 95% power to detect a difference from 6.0 to 3.9% in blood transfusion rates in favour of the anaemia arm, assuming an intracluster correlation coefficient of 0.005 and an average of 750 procedures per Trust per year (alpha = 0.05).

Both groups collected patient-level data on preoperative screening measures, treatment(s) received, operative details and postoperative outcomes including blood transfusions and SSI. These data were combined with routinely collected electronic data from each Trust’s Patient Administration System. Consent was obtained from executives and clinicians at each participating Trust before randomisation.

Health economic and process evaluations were conducted and will be reported separately.

### Intervention

Full details of how specific elements of the QIC methodology were implemented for this trial can be found in Supplementary File 1, but are summarised below with reference to terminology from the Cochrane Effective Practice and Organisation of Care taxonomy [39].

Following randomisation, participating teams from each Trust were invited to attend QIC events to learn about the initiative their Trust had been randomised to implement. Each of the two collaboratives was run and supported by a team of expert faculty with experience in the relevant clinical fields, improvement methodology and QICs (inter-professional education). Each collaborative consisted of a series of three, 1-day, face-to-face educational meetings (learning events) and a summative congress. Educational materials and games were used at learning events, where participating teams were taught about the respective clinical initiatives, and reviewed the evidence base, governance arrangements, business cases, communications strategy, data collection and reporting arrangements. A range of example materials were provided to teams. As the collaboratives progressed, teams developed and shared their own materials and presented on their own successes and challenges experienced as part of their improvement work. These were compared and discussed with others in the collaborative (monitoring of performance). Training on transferable quality improvement skills and time for teams to work on planning their improvement work were built into the learning event schedules. Cross-organisation working was encouraged at the learning events and throughout the collaboratives to develop communities of practice.

Between learning events (action periods), teams were encouraged to engage with local opinion leaders and undertake a local consensus process to implement change and the care pathways in their local setting. A continuous quality improvement approach (using the model for improvement) was encouraged and data were collected to measure the impact of changes (audit and feedback). To support this, regular feedback, support and reminders were provided to the teams by the faculty via email, webinars and regular coaching calls. Teams were required to submit a monthly written progress report. Performance data was presented within each of the collaboratives in a newsletter which included team rankings by data completion, to encourage engagement. A bespoke data collection system was created to collate manually collected data and routinely collected data held within local health information systems. This system automatically generated run charts allowing teams to track their progress over time (audit and feedback). Team progress was monitored by the faculty and support interventions were tailored to each team’s needs. Financial assistance was provided to teams to support attendance at learning events, dedicated staff time to work on the improvement initiatives and data collection. Product costs for intravenous iron (Ferrinject) and MSSA decolonising body wash and nasal gel (Octenidine) were provided when administered in line with previously published protocols, on a pay for performance basis dependant on data entry [40, 41].

The QICs ran from May 2018 to May 2019 (Table 1) including a 6-month implementation phase (May 2018–November 2018) during which it was expected that Trusts would develop and embed their preoperative pathways into usual care. A 12-month data collection period (November 2018–November 2019) followed; outcome data were collected for procedures performed during this time for trial analysis. During this period, it was expected preoperative pathways would be embedded, refinements made, and sustainability planning would take place. To minimise the risk of contamination or resentful demoralisation between the two trial arms, all Trusts were given the opportunity to join a new QIC, focussing on the pathway they had not implemented as part of the trial, after the trial data collection period was over.

**Table 1:**
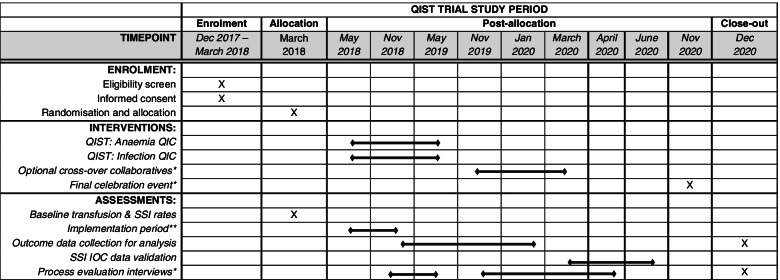
Standard Protocol Items: Recommendations for Interventional Trials (SPIRIT) figure for the Quality Improvement for Surgical Teams (QIST) trial as delivered

*QIC* quality improvement collaborative, *SSI* surgical site infection, *IOC* independent outcomes committee

*The timeline involved towards the end of the QIST programme was adversely affected by the COVID-19 pandemic. The timing and nature of the cross-over collaborative learning events and final celebration events changed in light of national guidance. The cross-over collaboratives consisted of 2 learning events (Nov 2019 and March 2020); a third had been planned but was cancelled due to lockdown restrictions. The final celebration event was delayed until November 2020, and this was held virtually instead of in person as originally planned

**The implementation period was a 6-month period during which it was expected Trusts would develop and implement their preoperative pathways before the trial measurement/outcome data collection phase began

### SSI data validation

To standardise the classification of SSI, an Independent Outcome Classification (IOC) group of orthopaedic surgeons experienced in revision arthroplasty was convened. They reviewed the case notes for all records flagged as possible SSI by the participating teams and applied Public Health England (PHE) and Centre for Disease Control (CDC) criteria for deep (up to 90 days post-surgery) and superficial (up to 30 days) SSI to all cases and a consensus reached for final trial outcomes. Deep SSI caused by MSSA as defined by the PHE definition up to 90 days post-surgery was the primary outcome for the infection arm. We had planned to include a consultant microbiologist in the IOC, but a lack of availability due to the COVID-19 pandemic meant the makeup of the IOC had to change.

### Statistical analysis

Analyses were undertaken in Stata v15 (StataCorp, Texas) using two-sided statistical tests at the 5% significance level. Unless otherwise stated, Trusts were assumed to have implemented their assigned protocol only and, as such, patients and procedures were analysed in the groups to which their treating Trust was randomised; however, not all randomised Trusts, or associated procedures, could be included in the analyses and so missing data were removed from the analysis and available-case analysis was used (each outcome was analysed using only available data). It was not possible to analyse data from those 14 Trusts that were randomised but did not provide any data, and missing data within participating Trusts was not imputed. Data were received and analysed at the procedure-level, rather than the patient-level, since it was possible that patients could undergo more than one eligible procedure during the study period. We anticipated this number would be small and so treated procedures as independent. A statistical analysis plan created prior to the analysis was agreed with the trial oversight committee.

Co-primary outcomes were both analysed using mixed effects logistic regression models, adjusted for procedure type (hip or knee), age and sex of the patient, and the Trust-level factors that were used for randomisation: number of hip and knee replacements performed 2016/2017 (in continuous form), and the LfM league indicators. Trust was included as a random effect. All of the secondary outcomes related to postoperative SSI, readmissions and critical care admissions were analysed in the same way as the primary outcomes. Length of hospital stay was analysed using a Poisson regression model, and length of critical care stay was analysed using a Poisson model when just including those with a stay on critical care, and zero-inflated Poisson regression after imputing zero days on critical care when there was no admission to critical care — all were adjusted in the same way as the primary analyses.

Sensitivity analyses were planned to rerun both primary models excluding any Trusts that implemented the opposite protocol to the one they were assigned (deemed to be contaminated) and also to include ischaemic heart disease as a covariate in the analysis of the anaemia primary outcome as this is often influential in determining if a patient requires a blood transfusion. Three additional sensitivity analyses were undertaken post hoc to account for any possible underreporting of the outcomes by sites. These analyses excluded sites that:

- Reported no transfusions, from the anaemia primary analysis;
- Reported no infections, from the infection primary analysis; and
- Had all reported infections confirmed by the IOC, from the infection primary analysis.

All results are presented as the relevant effect estimate (odds ratio (OR) or incident rate ratio (IRR)), 95% confidence interval (CI) and *p*-value.

### Deviations from protocol

As the trial progressed, some changes to the planned delivery of the collaboratives were made. Additional support was made available as part of the intervention, in particular participating teams were assigned a senior faculty coach who offered a programme of regular coaching calls for the duration of the collaboratives. Information from these coaching calls, along with the monthly written reports, was used to tailor support to each team. In addition, funding was made available to support attendance at learning events for those travelling more than four hours each way, to support dedicated staff time to work on the improvement initiatives, for data collection and some product costs (Supplementary File 1). Due to the global coronavirus pandemic and national restrictions, the cross-over collaboratives consisted of only two learning events, rather than three; the final celebration event was delayed and ultimately held virtually.

## Results

### Recruitment

**Fig. 1:**
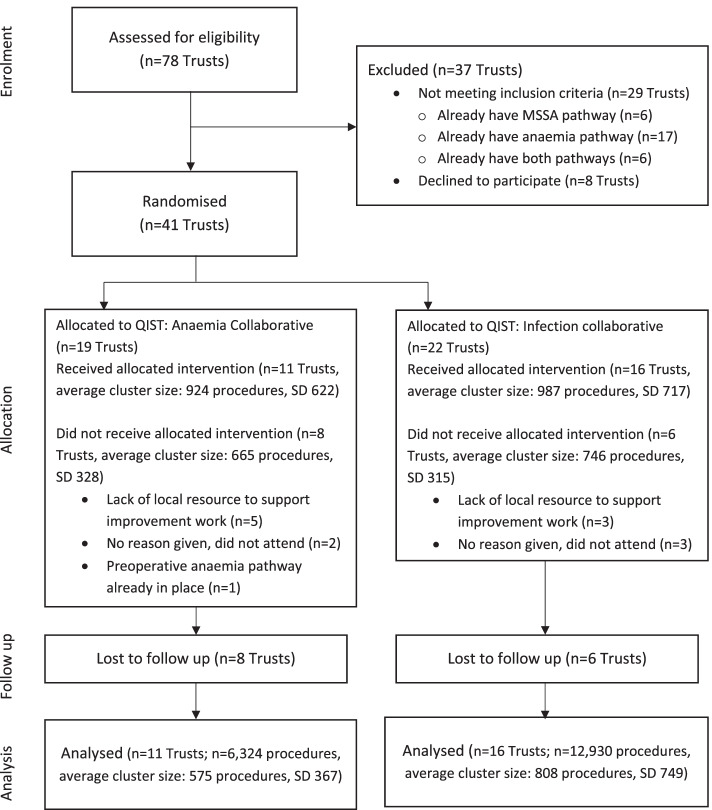
CONSORT flow diagram

Trusts were recruited between December 2017 and March 2018. Seventy-eight Trusts indicated that they were interested in participating. Following application of the eligibility criteria, 41 Trusts were randomised, in March 2018: 19 to QIST: Anaemia (46.3%) and 22 (53.7%) to QIST: Infection (Fig. 1). Post-randomisation, Research and Development departments were asked to sign the required agreements and confirm they had the capacity and capability within their Trust to participate in this trial. Capacity and capability was confirmed and a green light to participate was issued for 27 of the 41 Trusts (65.9%): 11 Anaemia (57.9% of 19 randomised) and 16 Infection (72.7% of 22 randomised) — it was not possible to collect data for trial analysis from Trusts who did not confirm capacity and capability to participate in the trial. Due to the way the intervention was implemented, all Trusts were required to be recruited at the same time and it was not possible to recruit additional Trusts after the collaborative intervention had started.

### Baseline data

Of the 27 participating trusts, the average number of procedures performed in 2016/2017 was 957 (SD 670), and half (*n* = 13, 48.1%) were awarded a good or outstanding LfM ranking (Table 2). Characteristics were well balanced across the two arms.

**Table 2:** Baseline characteristics of the Trusts as randomised (*n* = 41) and as included in the analysis (*n* = 27)

	As randomised^a^		As analysed^a^		
	Anaemia (*n* = 19)	Infection (*n* = 22)	Anaemia (*n* = 11)	Infection (*n* = 16)	Overall (*n* = 27)
**Number of THR and TKR undertaken in 2016/2017**					
Mean (*SD*)	814.7 (523.6)	916.4 (636.8)	924.0 (622.0)	980.4 (720.1)	957.4 (669.9)
Median (min, max)	669 (353, 2442)	661.5 (354, 2876)	707 (416, 2442)	661.5 (392, 2876)	671 (392, 2876)
**Dichotomised,*n*(%)**					
≤660	9 (47.4)	11 (50.0)	4 (36.4)	8 (50.0)	12 (44.4)
>660	10 (52.6)	11 (50.0)	7 (63.6)	8 (50.0)	15 (55.6)
**Learning from mistakes category**^**b**^**,*n*(%)**					
Outstanding	1 (5.3)	2 (9.1)	0 (0.0)	1 (6.3)	1 (3.7)
Good	8 (42.1)	9 (40.9)	5 (45.5)	7 (43.8)	12 (44.4)
Significant concern	7 (36.8)	7 (31.8)	4 (36.4)	6 (37.5)	10 (37.0)
Poor	3 (15.8)	4 (18.2)	2 (18.2)	2 (12.5)	4 (14.8)

^a^Differences between as randomised and as analysed are due to some Trusts not confirming capacity and capability

^b^Outstanding levels of openness and transparency, good levels of openness and transparency, significant concerns about openness and transparency, and poor reporting culture

### Procedures

A total of 25,066 (9860 Anaemia, 15,206 Infection) THR and TKRs were undertaken at the 27 Trusts over the 1-year period (1 November 2018 and 31 October 2019). Data were provided for 19,254 procedures: 6324 (32.8% of 19,254) in the anaemia arm (average per Trust 574.9, *SD* 366.7, median 458) and 12,930 (67.2% of 19,254) in the infection arm (average per Trust 808.1, SD 749.3, median 630).

In the time frame, 18,449 (97.9%) patients had one eligible procedure, 401 (2.1%) had two and 1 (0.01%) had three. Amongst all procedures, there was an almost equal split of THR (48.9% anaemia arm, 48.2% infection arm) and TKR procedures (51.1% anaemia, 51.8% infection). On average, at the time of their procedure, patients were 69.2 years old (*SD* 10.5). There were more females (59.0%) than males (41.0%). The most common comorbidity was hypertension (50.4%), followed by obesity (23.5%) and non-insulin-dependent diabetes mellitus (13.1%). Characteristics appeared balanced between the two arms (Table 3).

**Table 3:** Patient and procedure details

	Anaemia (***n*** = 6324)	Infection (***n*** = 12,930)	Total (***n*** = 19,254)
**Age, years**			
Mean (*SD*)	69.7 (9.9)	68.9 (10.8)	69.2 (10.5)
**Sex, *n*(%)**			
Male	2604 (41.2)	5297 (41.0)	7901 (41.0)
Female	3720 (58.8)	7633 (59.0)	11,353 (59.0)
**Procedure type, *n*(%)**			
Hip	3181 (50.3)	6238 (48.2)	9419 (48.9)
Knee	3143 (49.7)	6692 (51.8)	9835 (51.1)
**Comorbidities**	***n*= 6138**	***n*= 12295**	***n*= 18433**
Hypertension	3213 (52.3)	6080 (49.5)	9293 (50.4)
Non-insulin-dependent diabetes mellitus	780 (12.7)	1638 (13.3)	2418 (13.1)
Chronic ischaemic heart disease	595 (9.7)	1139 (9.3)	1734 (9.4)
Hypothyroidism	596 (9.7)	1105 (9.0)	1701 (9.2)
Atrial fibrillation	517 (8.4)	866 (7.0)	1383 (7.5)
History of circulatory disease	434 (7.1)	938 (7.6)	1372 (7.4)
COPD	397 (6.5)	694 (5.6)	1091 (5.9)
Rheumatoid arthritis	197 (3.2)	435 (3.5)	632 (3.4)
Alzheimer’s disease	17 (0.3)	43 (0.3)	60 (0.3)
Hyperthyroidism	23 (0.4)	34 (0.3)	57 (0.3)
Insulin-dependent diabetes mellitus	12 (0.2)	35 (0.3)	47 (0.3)
Dementia	16 (0.3)	21 (0.2)	37 (0.2)
Obesity	1479 (24.1)	2292 (23.1)	3771 (23.5)
Hypercholesterolemia	609 (9.9)	1062 (10.7)	1671 (10.4)
Smoking	327 (5.3)	632 (6.4)	959 (6.0)
Low sodium	106 (1.7)	193 (1.9)	299 (1.9)
Psoriatic arthritis	18 (0.3)	52 (0.5)	70 (0.4)

### Processes

The QIC approach used in this trial was intentionally pragmatic in terms of diagnostic criteria for anaemia (and iron deficiency) and treatment decisions for anaemia and MSSA decolonisation. This inevitably led to variation in how the two pathways were implemented at each site. A summary of key differences can be found in Table 4 with further detail provided in Supplementary Files 2 and 3.

**Table 4:** Summary of variation in approach to implementing anaemia screening and MSSA decolonisation pathways, rates of implementation and primary outcome reporting

Trial arm	Approach to diagnosing anaemia or MSSA decolonisation	Total number of procedures performed during trial 12-month measurement period (*n* range)	Procedures where records were provided (included in QIST analysis) (% range of total procedures performed)	Procedures for which pre-op pathway was implemented as part of QIST^a^ (% range of total procedures performed)	Reported potential SSIs, *n*	All SSIs confirmed by IOC^b^ (% range of reported potential SSIs)	IOC confirmed deep SSIs by causative organism (% range of procedures included in QIST)	Procedures requiring blood transfusion (% range)
Anaemia	Variation in Hb thresholds for diagnosing anaemia was observed. Lower limits ranged from 105 to 115g/L and upper limits ranged from 115 to 129g/L. Not all Trusts used sex-dependant thresholds	397–2805	20.4–100%	16.4–98.1	0–25	11–100%	MSSA: 0–0.7% Any^c^: 0–1.1%	0–11.6%
Infection	6 Trusts screened and only decolonised MSSA-positive patients. MSSA-positive rate ranged from 21.9 to 31.2% 10 Trusts decolonised all patients without screening	457–2786	22.6–100%	0–94.1%	0–75	9–100%	MSSA: 0–0.7% Any^c^: 0–2.1%	0–7.7%

Further details are provided in Supplementary Files 2 and 3

^a^During 12-month trial measurement period ^b^using either CDC or PHE definitions for deep or superficial SSI ^c^including MSSA

#### Anaemia

Trusts in the anaemia arm screened patients for anaemia via preoperative blood tests, performed a median of 58 days before surgery. Preoperative haemoglobin (Hb) levels were available for 6306 (99.7%) of the 6324 procedures (males: mean 143.3 g/L, *SD* 12.6; females: mean 131.3, *SD* 11.3), and ferritin for 93.2% (*n* = 5894; males: 173.1, *SD* 170.5; females: 103.2, *SD* 109.4).

The WHO criteria for diagnosing anaemia are the most commonly used worldwide (Hb < 120 females, < 130 males) [42]. As seen in Table 4 and Supplementary File 3, some Trusts used lower thresholds (i.e. Hb 120 for both sexes) to define anaemia and decide treatment. This means some patients who would typically be considered anaemic (i.e. males with Hb 125) were not by some Trust’s pathways. When the WHO definitions for anaemia are applied to our data, 885 (14.0%) patients are identified as having preoperative anaemia, of which 546 (61.7%) were accompanied by iron deficiency (ferritin < 100 μg/L).

A total of 5770 procedures (91.2%) are recorded as receiving no treatment, including 48.7% (431 of 885) of those identified as being anaemic by the WHO criteria. Intravenous iron was administered in 72 (1.1%) cases (all of which were anaemic); oral iron in 297 (4.7%) cases (of which 260 were anaemic); and 185 (5.1%) (of which 122 were anaemic) were referred for further investigation prior to surgery.

**Fig. 2:**
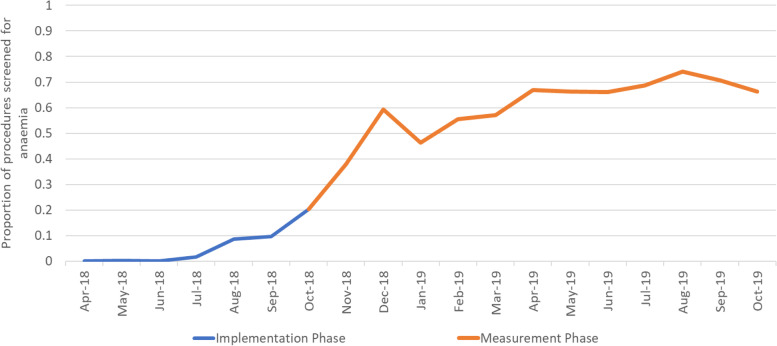
Implementation of the anaemia screening pathway over the 18-month QIST period: 6-month set up phase and 12-month data collection phase

Implementation of the anaemia screening pathway varied between Trusts; the proportion of procedures for which preoperative anaemia screening was performed during the 1-year measurement phase varied from 16.4 to 98.1%. Overall, 61.1% (6025 of 9860) of the total number of THR or TKR procedures performed across the 11 anaemia Trusts received preoperative anaemia screening. Figure 2 shows the proportion of the total number of THR or TKR procedures performed each month that received preoperative anaemia screening. This data spans both the implementation phase (pre-outcome data collection) and the measurement phase (1-year outcome data collection period). There was still an increasing trend in the first few months of the measurement phase (Oct–Dec 2018).

#### Infection

Trusts in the infection arm chose to implement MSSA decolonisation in one of two ways: screening all patients and decolonising only those who tested positive or routinely decolonising all patients without screening. Six of the 16 Trusts adopted a screening approach, resulting in 2768 procedures being screened preoperatively (21.4%). Of these, 893 (32.3%) tested positive for MSSA (Table 4 and Supplementary File 2).

A total of 12,019 decolonisation packs were dispensed for preoperative MSSA decolonisation as part of the QIST: Infection collaborative. The most common components were Octenisan body wash (89.6%, *n* = 10,768) and Octenisan nasal gel (68.0%, *n* = 8174). A total of 11,115 patients (92.5% of those dispensed) confirmed that they used the decolonisation pack that was provided to them.

**Fig. 3:**
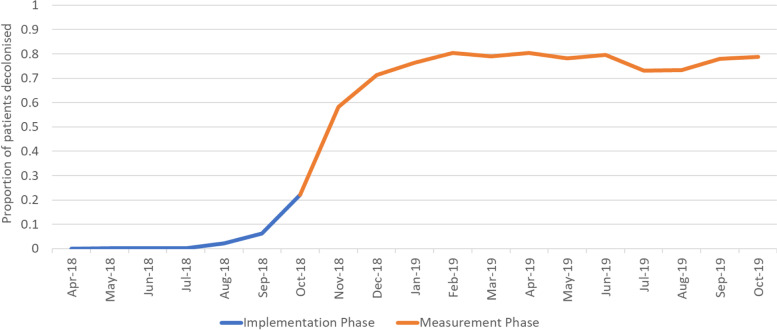
Implementation of the infection pathway over the 18-month QIST period: 6-month implementation phase and 12-month measurement phase

The correct implementation process for the infection arm differed depending on the approach to the pathway. For Trusts that screened patients, the correct implementation was to provide a nasal gel to patients who test positive, and not to those who test negative, whereas for the blanket approach, correct implementation was taken as confirmation that a nasal gel was issued. The implementation for the 1-year measurement phase varied by Trust; one Trust provided no data on their process, so had 0% implementation. The lowest percentage of correct implementation (for Trusts who recorded data) was 20.5%, and the highest was 94.1%. Overall, 73.7% (11,208 of 15,206) of the total number of THR or TKR procedures performed across 16 infection Trusts received correct implementation. This was slightly higher in the ten Trusts that implemented a blanket approach (75.4%, 8723 of 11,568 procedures), than the six Trusts who choose to screen (68.3%, 2485 of 3638 procedures). The rate of implementation was still increasing in the first few months of measurement (Fig. 3).

### Outcomes

Overall, 485 (2.5%) patients required a blood transfusion within 7 days of surgery: 183 (2.9%) in the anaemia arm and 302 (2.3%) in the infection arm. The average number of units per transfusion was similar for the two groups (1.7 (*SD* 1.0) in the anaemia arm; 1.6 (*SD* 0.6) in the infection arm). There was no evidence of a difference in transfusions between the two arms (adjusted *OR* 0.83 (i.e. lower odds in infection arm), 95% *CI* 0.36 to 1.91, *p* = 0.67; Table 5). The trust-level intracluster correlation coefficient was estimated as 0.23 (95% *CI* 0.13, 0.37).

**Table 5:** Results of the primary and secondary outcomes and sensitivity analyses

Outcome, ***n*** (%)^**a**^	Unadjusted		Adjusted^**b**^	
	Anaemia (***n*** = 6324)	Infection (***n*** = 12,930)	***OR*** (95% ***CI***)	***p***-value
*Primary outcomes*				
Blood transfusion	183 (2.9)	302 (2.3)	0.83 (0.36, 1.91)	0.67
Deep SSI (MSSA) (PHE)	8 (0.13)	18 (0.14)	1.01 (0.42, 2.46)	0.98
*Secondary infection outcomes*				
Deep SSI (MSSA) (CDC)	8 (0.13)	18 (0.14)	1.01 (0.42, 2.46)	0.98
Deep SSI (MSSA) (either)	8 (0.13)	18 (0.14)	1.01 (0.42, 2.46)	0.98
Deep SSI (any) (PHE)	23 (0.36)	48 (0.37)	0.97 (0.57, 1.64)	0.91
Deep SSI (any) (CDC)	23 (0.36)	48 (0.37)	0.97 (0.57, 1.64)	0.91
Deep SSI (any) (either)	23 (0.36)	48 (0.37)	0.97 (0.57, 1.64)	0.91
Superficial SSI (MSSA) (PHE)	5 (0.08)	4 (0.03)	0.49 (0.13, 1.86)	0.29
Superficial SSI (MSSA) (CDC)	5 (0.08)	10 (0.08)	0.93 (0.25, 3.45)	0.91
Superficial SSI (MSSA) (either)	5 (0.08)	10 (0.08)	0.93 (0.25, 3.45)	0.91
Superficial SSI (any) (PHE)	16 (0.25)	33 (0.26)	1.11 (0.55, 2.25)	0.77
Superficial SSI (any) (CDC)	18 (0.28)	48 (0.37)	1.30 (0.62, 2.73)	0.49
Superficial SSI (any) (either)	18 (0.28)	48 (0.37)	1.30 (0.62, 2.73)	0.49
*Secondary outcomes*				
	***n*= 6138**	***n*= 9911**		
Unplanned readmissions	390 (6.4)	464 (4.7)	1.09 (0.54, 2.19)	0.81
	***n*= 6324**	***n*= 12,693**		
Critical care admission	182 (2.9)	465 (3.7)	1.05 (0.46, 2.43)	0.90
			***IRR*(95%*CI*)**	***p*-value**
Length of stay on critical care^c,d^				
*N, mean (SD)*	182, 1.7 (1.4)	465, 1.5 (1.3)	1.05 (0.79, 1.41)	0.73
*Median (min, max)*	1 (0, 12)	1 (0, 17)		
*N, mean (SD)*	6324, 0.05 (0.4)	12,693, 0.06 (0.4)	0.97 (0.86, 1.09)	0.56
*Median (min, max)*	0 (0, 12)	0 (0, 17)		
	***n*= 6138**	***n*= 9911**		
Length of hospital stay^c^				
*Mean (SD)*	183 (3.9)	184 (3.1)	1.03 (0.93, 1.14)	0.53
*Median (min, max)*	3 (0, 137)	3 (0, 91)		
*Sensitivity analyses*			***OR*(95%*CI*)**	
*Excluding contaminated sites*	***n*=6324**	***n*=8607**		
Blood transfusion	138 (2.9)	230 (2.7)	0.81 (0.36, 1.84)	0.62
Deep SSI (MSSA) (PHE)	8 (0.13)	14 (0.16)	1.28 (0.52, 3.16)	0.59
*Ischaemic heart disease as covariate*	***n*=6138**	***n*=12,295**		
Blood transfusion	176 (2.9)	265 (2.2)	0.83 (0.37, 1.86)	0.65
*Excluding sites with no transfusions*	***n*=6243**	***n*=11,965**		
Blood transfusion	183 (2.9)	302 (2.5)	1.03 (0.52, 2.05)	0.94
*Excluding sites with no reported infections*	***n*=6243**	***n*=11,712**		
Deep SSI (MSSA) (PHE)	8 (0.13)	18 (0.15)	1.28 (0.51, 3.24)	0.60
*Excluding sites with 100% infection confirmation*	***n*=5162**	***n*=11,903**		
Deep SSI (MSSA) (PHE)	6 (0.12)	14 (0.12)	0.85 (0.31, 2.37)	0.76

Deep infections measured up to 90 days post-surgery; superficial infections measured up to 30 days post-surgery; unplanned readmission within 30 days of discharge; critical care admissions, and time spent on critical care, up to 30 days post-surgery

*SSI* surgical site infection, *MSSA* methicillin-susceptible *Staphylococcus aureus*, *PHE* Public Health England, *CDC* Centre for Disease Control, *OR* odds ratio, *IRR* incidence rate ratio

^a^Unless mean (SD), median, minimum, maximum as stated

^b^Treatment effects associated with infection arm presented

^c^Midnights in hospital

^d^First analysis includes only those admitted to critical care, second also includes those not admitted to critical care imputing 0 midnights in critical care and using a zero-inflated model

There were 346 cases of potential SSI reported by participating Trusts. Of these, the IOC confirmed 158 as either deep or superficial SSI based on PHE and/or CDC criteria (Table 5). Seventy-one (0.37%) were confirmed as deep SSIs (PHE criteria) caused by any organism within 90 days of surgery (23 (0.36%) in the anaemia arm; 48 (0.37%) in the infection arm). Of these, 26 (0.14%) were confirmed as deep SSIs (PHE criteria) caused by MSSA within 90 days of surgery (8 (0.13%) in the anaemia arm; 18 (0.14%) in the infection arm). There was no evidence of a difference between the two arms (adjusted *OR* 1.01, 95% *CI* 0.42 to 2.46, *p* = 0.98). The trust-level intracluster correlation coefficient was negligible (< 0.0001, with similarly small 95% CI limits).

A breakdown of transfusion and SSI rate by type of procedure (THR or TKR) is provided in Table 6.

**Table 6:** Breakdown of transfusion and SSI outcomes by type of procedure, where procedure known

	QIST: Anaemia arm	QIST: Infection arm
Transfusion (*n*, %)		
THR	138/3175 (4.3)	179/6118 (2.9)
TKR	45/3135 (1.4)	123/6506 (1.9)
Deep SSI (MSSA *n*, (%))		
THR	4/3175 (0.1)	9/6118 (0.1)
TKR	4/3135 (0.1)	9/6506 (0.1)
Deep SSI (any organism *n*, (%))		
THR	15/3175 (0.5)	26/6118 (0.4)
TKR	8/3135 (0.3)	22/6506 (0.4)

None of the secondary outcomes showed a statistically significant difference between the trial arms (Table 5).

### Sensitivity analysis

Sensitivity analyses undertaken to exclude Trusts that were deemed to be contaminated showed that the results were robust. This result was mirrored in the analysis including ischaemic heart disease as an additional covariate.

Trusts were asked to report all potential cases of SSI for review by the trial IOC. As such, overreporting of potential SSIs, not all of which would be confirmed by the IOC, was expected. This was observed for most of the 27 participating Trusts; however, we found a large variation in the reporting of potential SSIs in the trial (Table 4 and Supplementary Files 2 and 3), with some Trusts (*n* = 4) reporting no suspected SSIs, and some Trusts (*n* = 5) having all of their suspected SSIs confirmed by the IOC, raising concerns of selective reporting. A similar degree of variation and possible underreporting was also apparent for the blood transfusion outcome (Table 4 and Supplementary Files 2 and 3). There were three Trusts that reported no patients requiring a blood transfusion. Due to this, it was decided post hoc to undertake sensitivity analyses, where the primary models were rerun excluding the sites that had reported no transfusions/infections. Results were robust (Table 5).

### Costs

Participating in the QIST collaboratives incurred a mean cost of £37,164 (*SD* 8446) per Trust for the 11 Trusts in the anaemia arm and £37,638 (*SD* 9850) for the 16 Trusts in the infection arm. Total costs for running the QICs were £395,618. As almost all point estimates for postoperative outcomes were the same or slightly worse in the respective intervention arms, there were no cost savings related to the measured outcomes to be realised.

## Discussion

Quality improvement collaborative methodology incorporates many facilitators for overcoming barriers to clinical guideline implementation. This large-scale trial of QICs found evidence that collaboratives may be successful in implementing change in preoperative elective orthopaedics in the NHS, though variation across Trusts was seen. However, despite implementation rates of 73.7% and 61.1% being achieved in the infection and anaemia arms respectively, improved patient outcomes were not observed. A high early dropout rate (14 of 41 randomised sites, 34.1%) and substantial variation in individual team engagement with the collaborative were also observed. These are important considerations for planning future collaboratives or trials in this area.

Collaborative methodology is typically pragmatic in how individual elements of an intervention are implemented locally. This is intended to improve local adoption and sustainability by allowing each team to consider their local circumstances, unknown to QIC organisers, when developing local pathways. This pragmatic approach was taken for QIST. For example, in the QIST: Anaemia collaborative, teams were advised on the merits of screening and the existing evidence base. However, we did not stipulate haemoglobin or ferritin thresholds for diagnosing anaemia or guiding treatment, nor did we stipulate the type, duration or timing of iron treatment. This resulted in variation in how preoperative anaemia pathways were implemented at different sites. This variation goes some way to explain why 48.7% of anaemic patients (defined by WHO criteria) received no treatment. This may have reduced the effect size of introducing anaemia pathways on postoperative outcomes. Similarly, in the QIST: Infection collaborative, teams were advised that 5 days of nasal decolonisation were required, ideally spanning the date of surgery. However, within the trial, there was no stipulation around the timing of decolonisation, or which products were used. This led to variation in how the pathways were implemented at each site, which in turn may have impacted the primary and secondary outcomes for this arm of the trial. Conversely, this pragmatic approach reflects the practical implementation of a pathway, so reports real-world effectiveness.

Previous quality improvement trials in the UK have highlighted the importance of incorporating dedicated time and resources for improvement at a local level and training to develop generic quality improvement capabilities [43, 44]. The QIST trial set out to address both of these elements. It has also been suggested that a narrower focus, for example implementing just one new initiative per improvement programme, might help local implementation and improve the chances of success [44]. It may be that our narrow focus contributed to the high adherence rates observed in some Trusts in this trial. However, this did not lead to high levels of engagement at all trial sites and substantial variation in adherence to pathways was still seen between sites, similar to a previous quality improvement study in the UK [44].

Although measures were taken to reduce the Hawthorne effect of taking part in a trial, such as including both arms as intervention arms, and providing the opportunity for teams to attend cross-over collaborative events at the end of the trial, it is possible there were uncontrollable effects of being in the QIST trial. For example, the outcome of blood transfusion can be affected by multiple factors, many of which are targeted by multimodal patient blood management programmes. It is possible that although teams in the control arm for QIST: Anaemia did not introduce preoperative anaemia screening, they may have reviewed other elements of their patient pathway as QIST had shone a light on the risks associated with blood transfusion. Examples may include Trusts reviewing their tranexamic acid policy, transfusion triggers, or increased caution in issuing transfusions as clinicians knew these were being measured. Similarly, there are many factors that influence SSIs such as practices around antibiotic prophylaxis, surgical technique and patient warming. Furthermore, we are aware that some Trusts did implement both measures during the trial measurement period, although we attempted to adjust for this in our sensitivity analyses. This is reflective of the complex systems in which patient care is delivered. In addition, as both arms received an intervention, it was not possible to compare outcomes to a no intervention control.

Our results also suggest that, on the whole, more time was required to implement these changes than we had allocated. We incorporated a 6-month implementation period into our trial design, but our data suggest a longer implementation period was needed and a 9- or 10-month period may have been more appropriate.

One of the main limitations of our study was that the trial under-recruited and the observed outcome event rates were much smaller than anticipated in the sample size calculations. Anticipated and observed control rates were 0.75% and 0.13%, respectively, for MSSA deep SSIs and 6% and 2.3%, respectively, for blood transfusions. It may be that outcomes have genuinely improved over time, in keeping with recent studies which question the effectiveness of preoperative anaemia management in contemporary practice [45, 46]. However, it is also possible that some of this was due to underreporting, as for many Trusts part of the challenge of this trial was to implement robust patient follow-up procedures, particularly around SSI surveillance. Whilst funding, support and standardised advice were provided on how to perform SSI surveillance and report possible SSIs, it is possible there was variation in how this follow-up was performed and/or reported between Trusts. The variation seen in SSI rates and the differences in the rate of reported to IOC confirmed SSIs may reflect variation in surveillance and/or reporting practices between Trusts.

## Conclusions

Quality improvement collaboratives did not result in improved patient outcomes in this trial; however, there was some evidence that they can support successful implementation of new preoperative patient pathways in the NHS.

## Supplementary Information

**Additional file 1.** Summary of how elements of the Institute for Healthcare Improvement Breakthrough Series Collaborative model were applied to the Quality Improvement for Surgical Teams (QIST) collaboratives.

**Additional file 2.** Variation in approach to implementing MSSA decolonisation pathway, rates of implementation and primary outcome reporting.

**Additional file 3.** Variation in approach to implementing preoperative anaemia pathway, rates of implementation and primary outcome reporting.

## Data Availability

The anonymised datasets used and/or analysed during the current study are available from the corresponding author on reasonable request.
