# Supplementary material for: Scaling up Quality Improvement for Surgical Teams (QIST)—avoiding surgical site infection and anaemia at the time of surgery: a cluster randomised controlled trial of the effectiveness of quality improvement collaboratives to introduce change in the NHS
Source: Implement Sci. 2022 Mar 12;17:22. doi: 10.1186/s13012-022-01193-9 (PMC8917366; doi:10.1186/s13012-022-01193-9)
Supplement: Supplementary file 1 — Additional file 1. Summary of how elements of the Institute for Healthcare Improvement Breakthrough Series Collaborative model were applied to the Quality Improvement for Surgical Teams (QIST) collaboratives. [file 13012_2022_1193_MOESM1_ESM.docx]

Supplementary File 1: Summary of how elements of the Institute for Healthcare Improvement Breakthrough Series Collaborative model were applied to the Quality Improvement for Surgical Teams (QIST) collaboratives

| **Elements of Collaborative** | **How quality improvement collaboratives were delivered during the QIST trial** |
| --- | --- |
| Topic Selection | 1. Real-world improvements seen with these MSSA and anaemia screening protocols 2. They are not yet widely used across the NHS |
| Faculty Recruitment | 1. CI (Mike Reed) and clinical lead (Ashley Scrimshire) both completed the Institute for Healthcare Improvement Breakthough Series College training in Boston, USA before commencement of the QIST collaboratives 2. In addition, a team of expert faculty were recruited and participated in the development and delivery of the QIST collaboratives. The faculty include:    1. QIST: Anaemia: Dr A Kotze (consultant anaesthetist), Dr C Tiplady (Consultant haematologist), A Marriott (Surgical care practitioner), J Turner (staff nurse), W Atkinson (staff nurse)    2. QIST: Infection: Dr D Tate (consultant microbiologist), Mr A Malviya (consultant orthopaedic surgeon),    3. SSI surveillance (relevant to data collection for both trial arms): T Lamagni (Public Health England, Head of Surgical Site Infection Surveillance Service), G Lowdon (Surgical site infection surveillance lead)    4. Quality improvement: Mrs A Laverty (Chief experience officer), Steve Harrison (Head of quality improvement, Sheffield Teaching Hospitals NHS Foundation Trust)    5. Clinical informatics: M Burgess (information analyst)    6. Patient representative: S Cadwallader |
| Enrolment of Teams | 1. Calls for interested centres through BOA and NHS Improvement to all NHS Trusts in England to senior leaders, management and clinicians to increase support and engagement 2. 41 Trusts randomised 3. All team members were healthcare professionals |
| Learning Sessions | 1. Learning events for the two trial arms were held on different dates for anaemia and MSSA collaboratives to avoid contamination. Before the first event teams were sent a welcome pack with background materials and some pre-work exercises. Three one day, face to face events took place in hotel conference suite facilities across England per collaborative. The dates and attendance for each of the learning events were:    1. QIST Anaemia       1. Learning event 1: 22^nd^ May 2018          1. Total 46 attendees from 11 teams. Mean per team 4.2 people (range 3-6)       2. Learning event 2: 27^th^ June 2018          1. Total 33 attendees from 9 teams. Mean per team 3.7 people (range 2-5)       3. Learning event 3: 11^th^ September 2018          1. Total 31 attendees from 11 teams. Mean per team 2.8 people (range 1-6)       4. Summative congress: 15^th^ May 2019          1. Total 31 attendees from 10 teams. Mean per team 3.1 people (range 2-4)    2. QIST infection       1. Learning event 1: 24^th^ May 2018          1. Total 62 attendees from 16 teams. Mean per team 3.9 people (range 1-6)       2. Learning event 2: 26^th^ June 2018          1. Total 48 attendees from 15 teams. Mean per team 3.2 people (range 1-6)       3. Learning event 3: 12^th^ September 2018          1. Total 50 attendees from 14 teams. Mean per team 3.6 people (range 1-7)       4. Summative congress: 16^th^ May 2019          1. Total 37 attendees from 14 teams. Mean per team 2.6 people (range 1-5) 2. Each event was attended by field experts, programme leads, improvement fellows, patient leaders and study team members from each participating Trust. Teams were offered 4 places at each learning event and multidisciplinary working was encouraged. Details of attendance at learning events can be found above. 3. The content of the learning events included training Trusts on the relevant protocol, reviewing the evidence base, governance arrangements, business cases, communications, pathways, data collection and reporting arrangements. Example materials were created and shared with the participating Trusts via a secure online file store. These included the documents above plus a range of promotional materials to raise the profile of their QIST work locally such as patient videos, posters, press release statements. Participating teams also generated their own materials, such as patient information leaflets and letters to primary care and shared these amongst the collaborative groups. Content of the learning events also included a substantial amount of generic QI skills training with the intention of supporting change and building QI capacity at sites. Details of the content of each learning event can be found in the supplementary materials. 4. A further series of three learning sessions at the end of the study period were run to teach all Trusts both interventions. These cross over collaborative events were also made available to Trusts not involved in the trial element of QIST. The timing and nature of the cross over collaborative learning events and final celebration event changed in light of the COVID-19 pandemic and national lockdown restrictions. The cross over collaboratives consisted of 2 learning events (12^th^ & 13^th^ Nov 2019 and 3^rd^ & 4^th^ March 2020). A third had been planned but was cancelled due to national restrictions. |
| Action Periods | 1. During the action periods participating teams worked on implementing change locally and collected data to measure the impact. 2. Financial support was provided to participating teams to support attendance at learning events for those travelling the furthest (>4 hours each way), to support dedicated staff time to work on the improvement initiatives and for data collection (one session of Band 6 time for 18 months for Trusts expected to perform up to 900 THR/TKRs per year, two sessions if >900 procedures, and three if >1800 procedures; plus 10 days of Band 6 data analyst time to perform Patient Administration System (PAS) data extractions and uploads). In addition, product costs for IV iron (Ferrinject) and MSSA decolonising body wash and nasal gel (Octenidine) were provided when administered to patients in line with previously published protocols (40,41). 3. In addition to faculty making themselves available to be contacted by teams, a programme of regular coaching calls between team members and project expert faculty, who were trained in coaching, was established and teams encouraged to take part. Each team was assigned the same coach for the duration of the collaborative. Teams also fed back regularly via written monthly reports and presenting at the learning sessions on their progress in implementing change. This data was regularly reviewed by the organizing faculty to aid individualization of the support offered to teams based on each team’s needs and preferences. A traffic light system was developed and maintained in an attempt to highlight to faculty where teams may have been struggling or disengaged with the process. |
| Summative Congress and Publications | 1. Summative congresses for the anaemia and infection collaboratives took place on 15^th^ & 16^th^ May 2019 respectively. 2. A final summative session was planned for September 2020. Due to COVID-19 this was delayed until Nov 2020 and delivered virtually instead of in person. 3. Protocol paper published and this paper reports the results. Results being presented at International Evidence and Implementation summit 2021 and British Orthopaedic Association annual congress 2021. 4. The QIST: Anaemia teams were actively involved in the development of national CQUIN target CCG10: Screening and treatment of iron deficiency anaemia in patients listed for major elective blood loss surgery”, for the year 2020-21. However, implementation of this target has been postponed due to the COVID-19 pandemic (47). |
| Measurement and Evaluation | 1. A bespoke, electronic data collection system was developed. This included manual data entry and uploading of PAS data from informatics teams. This system also incorporated a real time feedback mechanism that allowed participating teams to visualize and download their own data throughout the collaboratives. This allowed teams to track changes in their own process measures (i.e. screening rate) and outcomes (i.e. transfusion rate) over time, on a monthly basis. This was intended to support local implementation and provide benchmarking with peers. To aid data collection several prompts were built into the electronic data collection system using PAS data, for example if PAS data showed a patient were readmitted within 90 days the users were prompted to check and confirm if this was due to SSI or not. Teams were asked to report any potential SSI via the electronic system and an independent outcomes committee would then review each case and apply SSI criteria in a standardised fashion for definitive trial outcome. |

Reference

47. NHS England and NHS Improvement. Commissioning for quality and innovation (CQUIN): guidance for 2020 - 2021. 2020. Available from: https://www.england.nhs.uk/wp-content/uploads/2020/01/FINALCQUIN-20-21-Core-Guidance-190220.pdf. Cited 2021 May 25.
