# Supplementary material for: Scaling up Quality Improvement for Surgical Teams (QIST)—avoiding surgical site infection and anaemia at the time of surgery: a cluster randomised controlled trial of the effectiveness of quality improvement collaboratives to introduce change in the NHS
Source: Implement Sci. 2022 Mar 12;17:22. doi: 10.1186/s13012-022-01193-9 (PMC8917366; doi:10.1186/s13012-022-01193-9)
Supplement: Supplementary file 2 — Additional file 2. Variation in approach to implementing MSSA decolonisation pathway, rates of implementation and primary outcome reporting. [file 13012_2022_1193_MOESM2_ESM.docx]

Supplementary File 2: Variation in approach to implementing MSSA decolonisation pathway, rates of implementation and primary outcome reporting

| Trust | Approach to implementing MSSA decolonisation | Total number of procedures performed during trial 12-month measurement period | Procedures where records were provided (included in QIST analysis) (n, % of total procedures performed) | Procedures for which MSSA decolonisation pathway was implemented as part of QIST^a^ (n, % of total procedures performed) | Screening results, n (% of procedures screened) | Reported potential SSIs, n | All SSIs confirmed by IOC^b^ (n, % of reported potential SSIs) | IOC confirmed deep SSIs by causative organism (n, % of procedures included in QIST) | Procedures requiring blood transfusion (n, %) |
| --- | --- | --- | --- | --- | --- | --- | --- | --- | --- |
| 1 | Screen | 482 | 141 (29.3) | 137 (28.4) | MSSA positive = 44 (31.2)  MSSA negative = 97 (68.8) | 3 | 3 (100) | MSSA: 1 (0.7)  Any^c^: 3 (2.1) | 2 (1.4) |
| 2 | Screen | 457 | 457 (100.0) | 409 (89.5) | MSSA positive = 100 (21.9)  MSSA negative = 353 (77.2) | 18 | 2 (11) | MSSA: 0 (0.0)  Any^c^: 0 (0.0) | 12 (2.6) |
| 3 | Screen | 482 | 477 (99.0) | 380 (78.8) | MSSA positive = 146 (30.6)  MSSA negative = 322 (67.5) | 17 | 10 (59) | MSSA: 1 (0.2)  Any^c^: 2 (0.4) | 7 (1.5) |
| 4 | Screen | 589 | 582 (98.8) | 554 (94.1) | MSSA positive = 151 (26.0)  MSSA negative = 430 (73.9) | 9 | 4 (44) | MSSA: 2 (0.3)  Any^c^: 2 (0.3) | 34 (5.8) |
| 5 | Screen | 1107 | 1000 (90.3) | 859 (77.6) | MSSA positive = 299 (29.9)  MSSA negative = 672 (67.3) | 3 | 3 (100) | MSSA: 0 (0.0)  Any^c^: 1 (0.1) | 4 (0.4) |
| 6 | Screen | 521 | 158 (30.3) | 146 (28.0) | MSSA positive = 153 (96.8)^d^  MSSA negative = 1 (0.6) | 4 | 4 (100) | MSSA: 1 (0.6)  Any^c^: 2 (1.3) | 6 (3.8) |
| 7 | Blanket | 549 | 237 (43.2) | 0 (-) | N/A | 0 | 0 (-) | MSSA: 0 (0.0)  Any^c^: 0 (0.0) | 0 (0.0) |
| 8 | Blanket | 813 | 728 (89.5) | 692 (85.1) | N/A | 3 | 3 (100) | MSSA: 2 (0.3)  Any^c^: 3 (0.4) | 0 (0.0) |
| 9 | Blanket | 719 | 678 (94.3) | 675 (93.9) | N/A | 18 | 12 (67) | MSSA: 1 (0.2)  Any^c^: 6 (0.9) | 8 (1.2) |
| 10 | Blanket | 880 | 816 (92.7) | 769 (87.4) | N/A | 0 | 0 (-) | MSSA: 0 (0.0)  Any^c^: 0 (0.0) | 63 (7.7) |
| 11 | Blanket | 730 | 165 (22.6) | 150 (20.5) | N/A | 0 | 0 (-) | MSSA: 0 (0.0)  Any^c^: 0 (0.0) | 3 (1.8) |
| 12 | Blanket | 846 | 815 (96.3) | 785 (92.8) | N/A | 9 | 6 (67) | MSSA: 2 (0.3)  Any^c^: 3 (0.4) | 6 (0.7) |
| 13 | Blanket | 998 | 998 (100.0) | 928 (93.0) | N/A | 14 | 12 (86) | MSSA: 0 (0.0)  Any^c^: 6 (0.6) | 46 (4.6) |
| 14 | Blanket | 2786 | 2667 (95.7) | 2619 (94.0) | N/A | 75 | 24 (31) | MSSA: 4 (0.1)  Any^c^: 9 (0.3) | 70 (2.6) |
| 15 | Blanket | 504 | 504 (100.0) | 226 (44.8) | N/A | 17 | 13 (76) | MSSA: 0 (0.0)  Any^c^: 2 (0.4) | 36 (7.1) |
| 16 | Blanket | 2743 | 2507 (91.4) | 1879 (68.5) | N/A | 41 | 14 (34) | MSSA: 4 (0.2)  Any^c^: 9 (0.4) | 5 (0.2) |
| Total | - | 15,206 | 12,930 (85.0) | 11208 (73.7) | - | 231 | 110 (47.6) | MSSA: 18 (0.1)  Any^c^: 48 (0.4) | 302 (2.3) |
| Screen = Screening patients and only offering nasal MSSA decolonisation treatment to those testing MSSA positive. Blanket = Not screening, but giving all patients nasal MSSA decolonisation treatment. ^a^ during 12 month trial measurement period ^b^using either CDC or PHE definitions for deep or superficial SSI ^c^ including MSSA ^d^ This Trust only provided data for procedures where MSSA decolonisation was given after a positive screening test | | | | | | | | | |
