# Supplementary material for: Scaling up Quality Improvement for Surgical Teams (QIST)—avoiding surgical site infection and anaemia at the time of surgery: a cluster randomised controlled trial of the effectiveness of quality improvement collaboratives to introduce change in the NHS
Source: Implement Sci. 2022 Mar 12;17:22. doi: 10.1186/s13012-022-01193-9 (PMC8917366; doi:10.1186/s13012-022-01193-9)
Supplement: Supplementary file 3 — Additional file 3. Variation in approach to implementing preoperative anaemia pathway, rates of implementation and primary outcome reporting. [file 13012_2022_1193_MOESM3_ESM.docx]

Supplementary File 3: Variation in approach to implementing preoperative anaemia pathway, rates of implementation and primary outcome reporting

| Trust | Approach to implementing preoperative anaemia screening and treatment | | | | Total number of procedures performed during trial 12-month measurement period | Procedures where records were provided (included in QIST analysis) (n, % of total procedures performed) | Procedures for which pre-op anaemia screening pathway was implemented as part of QIST^a^ (n, % of total procedures performed) | Reported potential SSIs, n | All SSIs confirmed by IOC^b^ (n, % of reported potential SSIs) | IOC confirmed deep SSIs by causative organism (n, % of procedures included in QIST) | Procedures requiring blood transfusion (n, %) |
| --- | --- | --- | --- | --- | --- | --- | --- | --- | --- | --- | --- |
|  | Criteria used for diagnosing anaemia and iron deficiency to be treated with iron | Oral iron additional criteria and treatment regimen | IV iron additional criteria and treatment regimen | Criteria used for referral for further investigation of anaemia prior to surgery |  |  |  |  |  |  |  |
| 17 | M: Hb 115-129g/L  F: Hb 105 – 119g/L  & Ferritin: 12-100 & ESR <50 | 4 weeks ferrous sulfate 200mg TDS  Repeat bloods after 1 month | If eGFR <50 or intolerance or failure of oral iron to correct anaemia | Hb M < 115  F < 105 or Ferritin < 12  or ESR > 50 | 672 | 656 (97.6) | 611 (90.9) | 25 | 7 (28) | MSSA: 1 (0.2)  Any^c^: 3 (0.5) | 8 (1.2) |
| 18 | M: Hb 115-129g/L & Ferritin 40-300  F: Hb 105 – 119g/L & Ferritin 28-200 | eGFR >50  One month oral iron then check response. | eGFR <50 or intolerance or no increase of Hb with oral iron | Hb  <115 Male  <105 female | 2805 | 1474 (52.5) | 1451 (51.7) | 21 | 10 (48) | MSSA: 1 (0.1)  Any^c^: 5 (0.3) | 23 (1.6) |
| 19 | 105<Hb<130  & 30<Ferritin<200 | eGFR >50  Treatment “oral iron” type, dose and duration unknown | eGFR<50  Treatment “IV iron” type, dose and duration unknown | Hb<105 | 879 | 458 (52.1) | 442 (50.3) | 9 | 8 (89) | MSSA: 3 (0.7)  Any^c^: 5 (1.1) | 8 (1.8) |
| 20 | 105<Hb<119  Ferritin 15-100 | CRP ≤5 & eGFR ≥44  Check Hb response after 4 weeks. Continue until surgery if Hb improving | CRP >5 or eGFR <44  Or if Hb not increasing after 4 weeks of oral iron | Hb <105  or ferritin <15 | 398 | 322 (80.9) | 315 (79.1) | 14 | 6 (43) | MSSA: 0 (0.0)  Any^c^: 3 (0.9) | 11 (3.4) |
| 21 | M: Hb 115-129g/L & Ferritin 23-322  F: Hb 105 – 119g/L & Ferritin 11-291 | eGFR>50  28 day course ferrous sulphate. Bloods rechecked on admission for surgery. | eGFR<50  or surgery planned within 4 weeks. Bloods rechecked on admission for surgery. | M: Hb <115 or  323< ferritin <22  F: Hb<105 or  292< ferritin <10 | 849 | 738 (86.9) | 617 (72.7) | 3 | 3 (100) | MSSA: 0 (0.0)  Any^c^: 2 (0.3) | 31 (4.2) |
| 22 | M: Hb<130g/L  F: Hb<120g/L  & MCV ≤80 & ferritin <30 | Surgery 8+ weeks away  Oral iron for 4 weeks then recheck Hb. If Hb normalised continue oral iron until surgery. | Surgery <8weeks away  IV iron then recheck Hb in 2 weeks or on admission. If time available and Hb response, may need repeat IV iron dose after 2-4 weeks. | Consider if  Hb M < 130  F < 120  & MCV>80 & ferritin <30 | 757 | 697 (92.1) | 685 (90.5) | 11 | 1 (9) | MSSA: 0 (0.0)  Any^c^: 0 (0.0) | 27 (3.9) |
| 23 | M: Hb 115-129g/L  F: Hb 105 – 114g/L  &  12<Ferritin<100 & ESR<50  & “normal liver function” | eGFR ≥50mL/hr  Ferrous sulphate 200mg TDS for 4 weeks. Letter sent to patient. Repeat bloods after 1 month (if oral iron intolerant reduce to twice daily) | eGFR <50  Patient sent a letter. Bloods rechecked at pre-op assessment clinic. | Hb <115 Male  <105 female | 436 | 421 (96.6) | 403 (92.4) | 4 | 4 (100) | MSSA: 2 (0.5)  Any^c^: 2 (0.5) | 49 (11.6) |
| 24 | No pathway received | | | | 397 | 81 (20.4) | 65 (16.4) | 0 | 0 (-) | MSSA: 0 (0.0)  Any^c^: 0 (0.0) | 0 (0.0) |
| 25 |  |  |  |  | 503 | 386 (76.7) | 360 (71.6) | 5 | 2 (40) | MSSA: 0 (0.0)  Any^c^: 1 (0.3) | 2 (0.5) |
| 26 |  |  |  |  | 775 | 775 (100.0) | 760 (98.1) | 8 | 6 (75) | MSSA: 1 (0.1)  Any^c^: 2 (0.3) | 17 (2.2) |
| 27 |  |  |  |  | 1389 | 316 (22.8) | 316 (22.8) | 15 | 5 (33) | MSSA: 0 (0.0)  Any^c^: 0 (0.0) | 7 (2.2) |
| Total | - | | | | 9860 | 6324 (64.1) | 6025 (61.1) | 115 | 52 | MSSA: 8 (0.1)  Any^c^: 23 (0.4) | 183 (2.9) |
| M=Male, F=Female, Hb reported in g/L, ferritin reported in mcg/L, ESR reported in mm/hour, eGFR reported in ml/min, CRP = C-reactive protein reported in mg/L, IV = intravenous, TDS = Three times per day ^a^ during 12 month trial measurement period ^b^using either CDC or PHE definitions for deep or superficial SSI ^c^ including MSSA | | | | | | | | | | | |
